# Targeting the AKT‐P53/CREB pathway with epicatechin for improved prognosis of traumatic brain injury

**DOI:** 10.1111/cns.14364

**Published:** 2023-07-18

**Authors:** Ziheng Wang, Zhichao Lu, Yixun Chen, Chenxing Wang, Peipei Gong, Rui Jiang, Qianqian Liu

**Affiliations:** ^1^ Department of Neurosurgery Affiliated Hospital of Nantong University, Medical School of Nantong University Nantong China; ^2^ Research Center of Clinical Medicine Affiliated Hospital of Nantong University Nantong China; ^3^ Centre for Precision Medicine Research and Training, Faculty of Health Sciences University of Macau Macau China; ^4^ Eye Institute Affiliated Hospital of Nantong University, Medical School of Nantong University Nantong China

**Keywords:** epicatechin, inflammation, microglia, neurological function, traumatic brain injury

## Abstract

**Aims:**

The aim of this study was to evaluate the effect of epicatechin, on neurological recovery and neuroinflammation after traumatic brain injury (TBI) to investigate its potential value in clinical practice.

**Methods:**

TBI model was established in adult rats by CCI method. The effect of epicatechin was evaluated after intraperitoneal injection. Neurological recovery after TBI was assessed by Morris Water Maze, mNSS score, Rotarod test and Adhesive removal test. Protein and gene expression was assessed by Western blot, ELISA, PCR and immunofluorescence. Furthermore, the use of AKT pathway inhibitors blocked the therapeutic effects of epicatechin clarifying AKT‐P53/CREB as a potential pathway for the effects of epicatechin.

**Results:**

Administering epicatechin after TBI prevented neuronal death, reduced neuroinflammation, and promoted neurological function restoration in TBI rats. Network pharmacology study suggested that epicatechin may exert its therapeutic benefits through the AKT‐P53/CREB pathway

**Conclusion:**

These results indicate that epicatechin, a monomeric compound derived from tea polyphenols, possesses potent antioxidant and anti‐inflammatory properties after TBI. The mechanism may be related to the regulation of the AKT‐P53/CREB signal pathway.

## Graphical abstract

Epicatechin has therapeutic benefits via the AKT‐P53/CREB pathway by network pharmacology study. These results imply that epicatechin is a potentially effective therapy for neurological impairments following TBI.

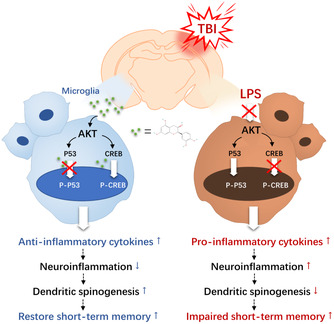

## 1 INTRODUCTION

Traumatic brain injury (TBI) is the most prevalent traumatic disease in neurosurgery and incurs significant treatment costs. Unfortunately, there are limited effective treatments available to significantly improve the prognosis of TBI patients, leading to high mortality and disability rates.^1^, ^2^ Excessive neuroinflammation is recognized as a critical contributor to the pathophysiology of TBI, although the precise mechanisms remain incompletely understood. TBI can trigger an amplified inflammatory cascade, resulting in cell death, blood–brain barrier disruption, and cerebral edema,^3^, ^4^ including activation of microglia and astrocytes, recruitment of leukocytes, and release of cytokines and chemokines. Thus, reducing excessive neuroinflammation following TBI is a crucial strategy for treating brain injury secondary to TBI.^4^, ^5^

One of the earliest manifestations of neuroinflammation is the rapid activation of microglia, leading to the release of inflammatory chemokines and upregulation of pro‐inflammatory cytokines such as interleukin‐1β (IL‐1β), interleukin‐6 (IL‐6), and tumor necrosis factor‐α (TNF‐α).^6^, ^7^ Studies suggest that inflammatory substances produced by activated microglia such as the interleukin family, tumor necrosis family, and interferon family can activate nuclear factor (NF)‐κB, protein kinase B (AKT), and signal transducer and activator of transcription (STAT) signaling pathways, which are cross‐regulated by various methods, including communication with other signaling pathways, resulting in persistent activation of inflammation‐relevant pathways. substances produced by activated microglia. Due to the persistent activation of these pathways,^8^ excessive levels of pro‐inflammatory cytokines and microglial cytotoxic mediators are produced, leading to excessive neuroinflammation in the TBI lesion site and poor prognosis for TBI patients.^6^ Therefore, reducing excessive neuroinflammation induced by microglia is essential to decrease psychiatric effects from TBI and enhance TBI patients' recovery.^9^, ^10^

Epicatechin is a monomeric molecule found in cocoa and green tea, derived from tea polyphenols, which possess hypotensive, hypolipidemic, and antioxidant properties.^11^, ^12^ Previous research has demonstrated its potent anti‐inflammatory and antioxidant effects in various neurological disease models such as Alzheimer's disease (AD) and Parkinson's disease, attributed to its good lipid solubility and ability to penetrate the blood–brain barrier (PD).^13^, ^14^ Additionally, studies have shown that epicatechin can prevent the production of Bax in PC12 cells induced by taurine deoxycholic acid,^15^ indicating that epicatechin may be useful in suppressing neuronal death. However, whether epicatechin has significant therapeutic effects on excessive neuroinflammation after TBI has not been explored.^16^ In this study, we evaluated the efficacy of epicatechin in inhibiting neuroinflammation after TBI and its potential biological mechanism of action using a rat model. Through network pharmacology, we aimed to identify drugs with therapeutic potential for TBI, thereby improving the prognosis and quality of life of TBI patients.

## 2 MATERIALS AND METHODS

### 2.1 Experimental animals

Adult male SD rats were provided by the Nantong University Animal Experiment Center. The animals were housed in filtered cages with automatic lighting, on a 12‐h light–dark cycle, and given regular feed.

### 2.2 Establishment of CCI models

Rats were anesthetized with isoflurane gas and placed on a stereotaxic machine. A median incision was made on the scalp, and a 2.0‐mm‐diameter bone window was created 1.0 mm posterior to the herringbone suture and 2.0 mm lateral to the median sagittal line. The hydraulic impact connector was placed in a small hole in the skull and sealed with dental resin while the dura remained intact. The parietal cortex was impacted by the hydraulic impact connector, connected to a hydraulic impact tool.

### 2.3 Drug administration

Epicatechin and AKT‐IN (Table S1) were dissolved in 10% DMSO and injected intraperitoneally. Four different doses of epicatechin (0.5, 1, 1.5, and 2 mg/kg) were administered orally once daily from the 1st to the 7th day after TBI. AKT‐IN was administered as directed by the manufacturer. LPS was added to the microglia medium at a concentration of 1 μg/mL.

### 2.4 Modified neurological severity score

To assess the neurological function in rats, the modified neurological severity score (mNSS) was assessed on days 1, 7, and 14 after TBI. The mNSS tests comprise a combination of motor, sensory, balance, and reflex assessments. A score of 1 point is assigned for each absent reflex or abnormal response. Thus, on a scale of 0–18, a score of 0 indicates normal neurological function without deficits, while a score of 18 indicates severe neurological deficits.

### 2.5 Morris water maze

The experiment had two phases: training and testing. The platform was placed in the middle of the circular pool's third quadrant. Mice were gently dropped into the pool from each of the four quadrants facing the pool's wall. The distance and time (escape latency) from entry to discovering the platform were recorded during the 4‐day training. On the 6th day, the platform was removed from the tank, and the rat was lowered into the pool at the same location where the original platform was. The rats' swimming path was then recorded for 2 min and traced using computer software during the testing phase.

### 2.6 Rotarod test

To assess the motor coordination of rats, an accelerated rotation test was conducted before TBI and on days 1, 7, and 14 after TBI using a rotarod apparatus. The rotarod spindle had a diameter of 10 cm and was accelerated from 4 to 30 rpm within 60 s, with a constant speed of 30 rpm held for 300 s. The time elapsed until the rat fell off the spindle was recorded with a sensor.

### 2.7 Adhesive removal test

To evaluate somatosensory function, a modified adhesive removal test was conducted. Two adhesive‐backed paper dots were applied to the wrist of each forelimb after the rats had been given time to get accustomed to the test environment. Then, the rats were returned to the testing cage where they usually removed the paper dots with their teeth. The time spent contacting and removing two stimuli per leg was recorded during the training, which was conducted five times a day for 3 days, and the rats that could remove both dots within 10 s in the experimental group were included in subsequent interventions and TBI model or sham surgery before the final adhesion removal test.

### 2.8 Hematoxylin and eosin (HE) staining

On day 7, TBI rats were anesthetized with isoflurane and perfused with saline and paraformaldehyde. After dehydration with a cryostat, two consecutive sections were obtained (Thermo Fisher Scientific) and gradually rehydrated before staining with hematoxylin for 10–15 min and counterstaining with 0.5% eosin for 2–5 min. Following dehydration in ethanol and xylene, the sections were mounted on glass slides for microscopic examination.

### 2.9 Immunofluorescence analysis

To retrieve the antigen, sections were incubated with an antigen retrieval solution (Solarbio) for 2 h. Next, primary antibodies were applied to cell or brain sections and incubated overnight at 4°C. The following day, the sections were incubated with a secondary antibody mixture for 1 h at 37°C. Morphological characteristics were evaluated by staining the samples with DAPI (Solarbio) for 40 min at 30°C and observing them under a fluorescence microscope (DM 5000B; Leica). The antibodies used for immunofluorescence are listed in the Materials and Methods section for antibodies.

### 2.10 TUNEL assay

The TUNEL assay was performed using the One‐Step TUNEL Apoptosis Assay Kit (Beyotime). The TUNEL reaction mixture (50 μL) was incubated at 37°C for 60 min. After cleaning with PBS, cells were observed using fluorescence microscopy.

### 2.11 Calcein AM/PI cytotoxicity assay

Microglia and astrocytes were inoculated on cell crawls and washed with PBS. The Calcein AM/PI assay solution was added in an appropriate volume and incubated at 37°C for 2 h. After incubation, the staining effect was observed under a fluorescent microscope protected from light.

### 2.12 Primary microglial cell culture

Following aseptic processing, primary glial cells were extracted from the cerebral cortex and injected into 25 cm^2^ culture flasks covered with 0.01% PLL after being digested with 0.25% trypsin at 37°C for 20 min. Cells were grown in whole F‐12 DMEM culture medium for 2 weeks, with the culture medium being replaced on the first day following the extraction and every 2 to 3 days thereafter. After full fusion, flasks were incubated for 30 min at 37°C and 180 rpm on a rotary shaker to separate microglia.

### 2.13 Western blotting

To investigate changes in protein expression in the injured portions of the rat brain/lateral cortex, tissue samples were collected at different time points. The proteins were separated using SDS‐PAGE and then transferred to 0.45 μm PVDF membranes. The membranes were blocked with 5% bovine serum albumin for 2 h and then incubated with primary antibodies (listed in Table S1) overnight. The following day, the membranes were incubated with secondary antibodies (listed in Table S1) at room temperature for 1 h (Billerica Millipore). Protein band signals were detected using the Chemidoc detection system (Bio‐Rad) and quantified using ImageJ software.

### 2.14 Enzyme‐linked immunosorbent assay (ELISA)

The protein levels of IL‐1β, IL‐4, TNF‐α, and IL‐10 (Sabbiotech) were quantified in tissues or cultured cells using ELISA kits according to the manufacturer's instructions (Table S1).

### 2.15 Quantitative real‐time polymerase chain reaction (qRT‐PCR)

To determine mRNA expression levels, 2 μg of RNA from each sample was reverse transcribed using the PrimeScript TMRT kit (Takara Bio Inc). Quantitative reverse transcription polymerase chain reaction (qRT‐PCR) was performed on an ABI QuantStudio5 Q5 real‐time PCR System (Thermo Fisher Scientific) using SYBR® Green Master (Roche). The relative mRNA expression was evaluated and normalized to the expression of glyceraldehyde 3‐phosphate dehydrogenase (GAPDH) using the 2^−ΔΔCT^ method. The primer sequences used for PCR are provided below:

**Table:** 

Gene	Forward primer (5′‐3′)	Reverse primer (5′‐3′)
iNOS	CAGCTGGGCTGTACAAACCTT	CATTGGAAGTGAAGCGTTTCG
CSF1R	GCAGTACCACCATCCACTTGTA	GTGAGACACTGTCCTTCAGTGC
Argl	GAACACGGCAGTGGCTTTAAC	TGCTTAGCTCTGTCTGCTTTGC
P2RY12	CTTCGTTCCCTTCCACTTTG	AGGGTGCTCTCCTTCACGTA
GAPDH	ATGACCACAGTCCATGCCATC	GAGCTTCCCGTTCAGCTCTG

### 2.16 Phagocytosis assay

Microglia phagocytosis was assessed using the Phagocytosis Assay Kit (Cayman, USA) following the manufacturer's instructions.

### 2.17 Epicatechin target prediction

SDF files containing 3D structural data were obtained from the PubChem database for both 2D and 3D structures of epicatechin. Using the 3D structural information and the SwissTargetPrediction analytical tool, target locations for epicatechin were predicted.

### 2.18 TBI target screening

The GeneCards database (https://genealacart.genecards.org/) and the OMIM database (https://www.omim.org/) were searched for disease‐related targets using the term “Traumatic brain injury”. The results were combined to generate a list of TBI‐related targets.

### 2.19 Protein–Protein Interaction Network (PPI) construction

Targets for epicatechin and TBI were intersected to identify potential targets for epicatechin's defense against TBI. The analysis was restricted to *Homo sapiens*, and the resulting targets were imported into the STRING online service to construct the PPI network.

### 2.20 GO and KEGG enrichment analysis

The intersection targets of TBI were subjected to GO functional enrichment analysis using the Bioconductor bioinformatics package of R language software.

### 2.21 Statistical analysis

All data were analyzed using GraphPad Prism 9.1 and presented as mean ± SEM. Unpaired Student's *t*‐test, one‐way ANOVA, and two‐way ANOVA were used to compare the data. A *p*‐value of <0.05 was considered statistically significant. All experiments were repeated at least three times.

## 3 RESULTS

### 3.1 Molecular structure of epicatechin and study experimental design

**FIGURE 1:**
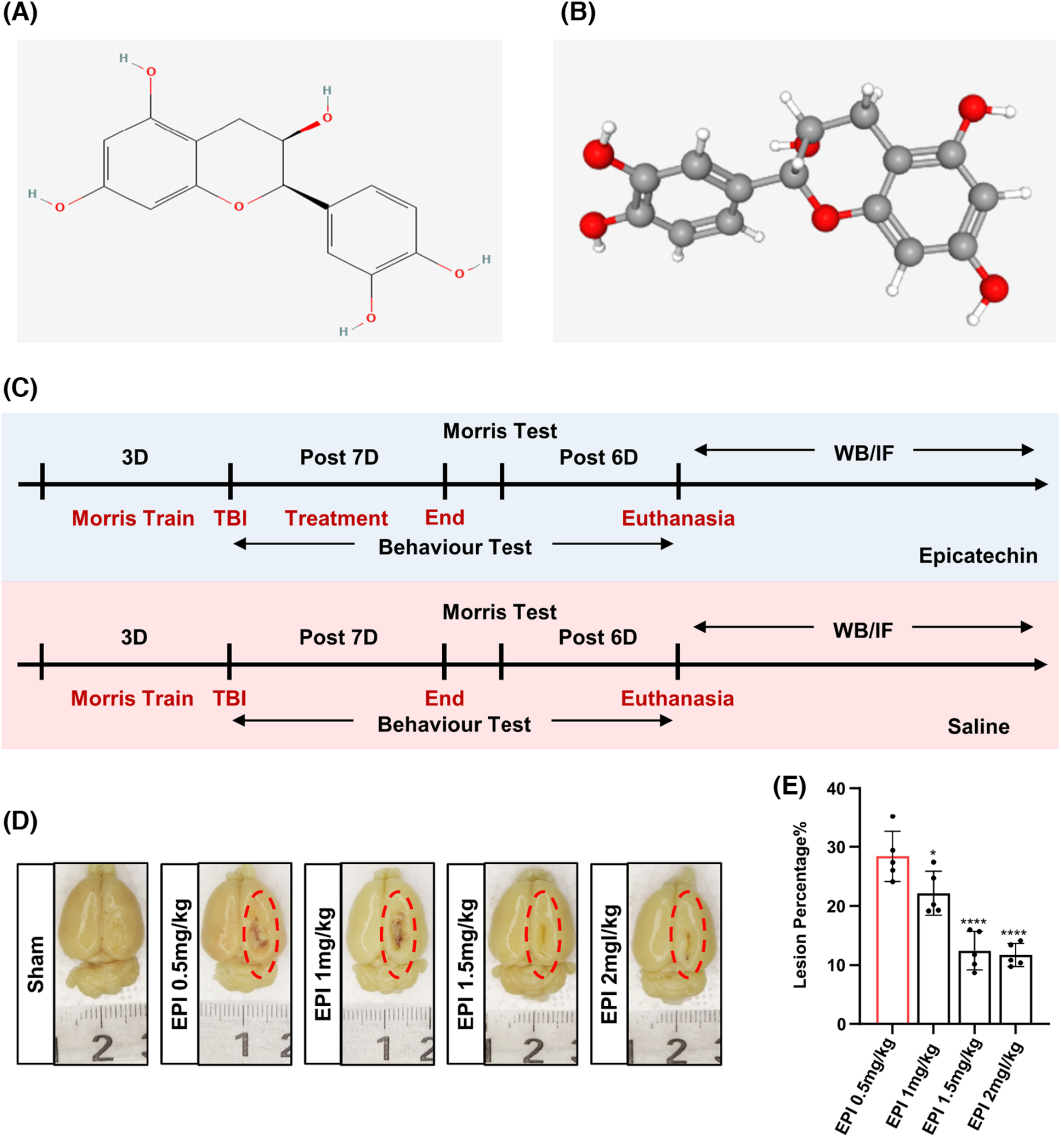
Epicatechin promotes brain tissue repair after TBI. (A, B) 2D and 3D structures of epicatechin retrieved from PubChem database; (C) Overview of the experimental procedure; (D, E) Representative results of intraperitoneal injections with different concentrations of epicatechin after TBI modeling and detection of the damaged area. ns, *p* > 0.05; *, *p* ≤ 0.05, **, *p* ≤ 0.01; ***, *p* ≤ 0.001;****, *p* ≤ 0.0001. The results are presented as mean ± SEM *n* = 5/group.

Epicatechin is a monomeric molecule derived from tea polyphenols, and it possesses beneficial anti‐oxidant, hypotensive, and hypolipidemic properties. The two‐dimensional and three‐dimensional chemical structures of epicatechin are presented in Figure 1A,B, respectively. Previous studies have shown that epicatechin exhibits good therapeutic properties against several models of neurodegenerative diseases, including Alzheimer's and Parkinson's disease. However, its therapeutic effect on excessive neuroinflammation and neurological decline after TBI remains unknown. Therefore, we designed a series of in vitro experiments to investigate the therapeutic effect and mechanism of epicatechin in a rat model of TBI (Figure 1C). We first explored the optimal dose of epicatechin for in vivo administration, and after testing 50 μg/100 g, 100 μg/100 g, 150 μg/100 g, and 200 μg/100 g for intraperitoneal injections, we found that a concentration of 150 μg/100 g was optimal for promoting the repair of the damaged region (Figure 1D,E).

### 3.2 Epicatechin promotes TBI wound healing and inhibits neuronal apoptosis

**FIGURE 2:**
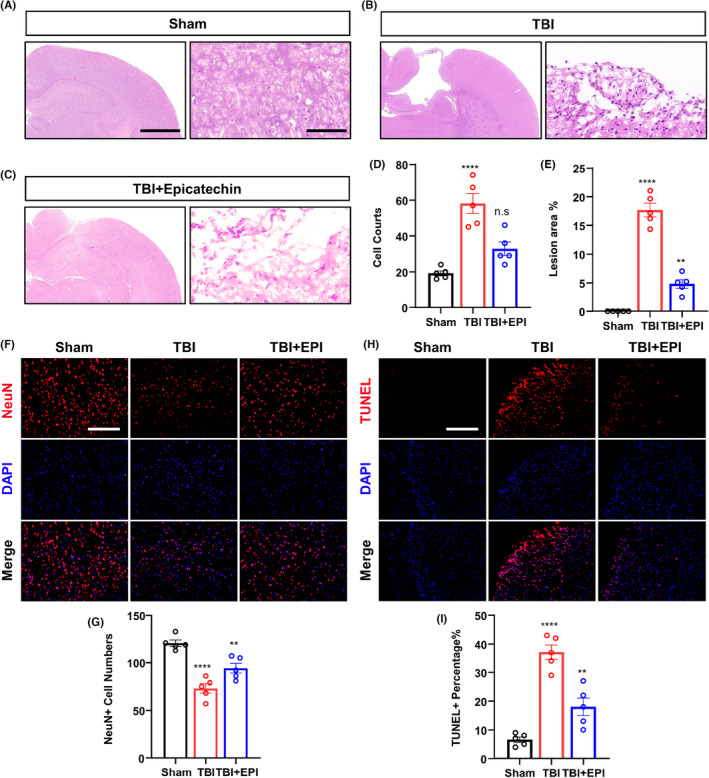
Epicatechin attenuates neuronal apoptosis after TBI. (A–C) Representative HE staining images of brain injury area after epicatechin treatment; (D) Quantitative analysis of infiltrating inflammatory cells in the injury area; (E) Quantitative analysis for the area of the injured region; (F) Representative immunofluorescence images of neurons in the injury area; (G) Quantitative analysis of the number of neurons in the injury area; (H) Representative images of TUNEL staining in the injury area; (I) Quantitative analysis of the proportion of TUNEL‐positive cells. ns, *p* > 0.05; *, *p* ≤ 0.05, **, *p* ≤ 0.01; ***, *p* ≤ 0.001;****, *p* ≤ 0.0001. The results are presented as mean ± SEM *n* = 5/group.

To assess the effect of epicatechin on TBI wound healing and neuronal apoptosis, we selected brain sections of SD rats on the 7th day after injury for HE staining. Epicatechin significantly promoted the healing of TBI wounds, and the number of infiltrated inflammatory cells in the wound decreased significantly after epicatechin treatment (Figure 2A–E). We also detected the number of neurons and apoptotic cells in the injury area by immunofluorescence and TUNEL staining. The results showed that the number of neurons was significantly reduced and the number of apoptotic cells was significantly increased after TBI. However, the administration of epicatechin could rescue the decrease in neuron number and inhibit apoptosis in the injury area (Figure 2F–I).

### 3.3 Epicatechin improves learning and memory functions after TBI

**FIGURE 3:**
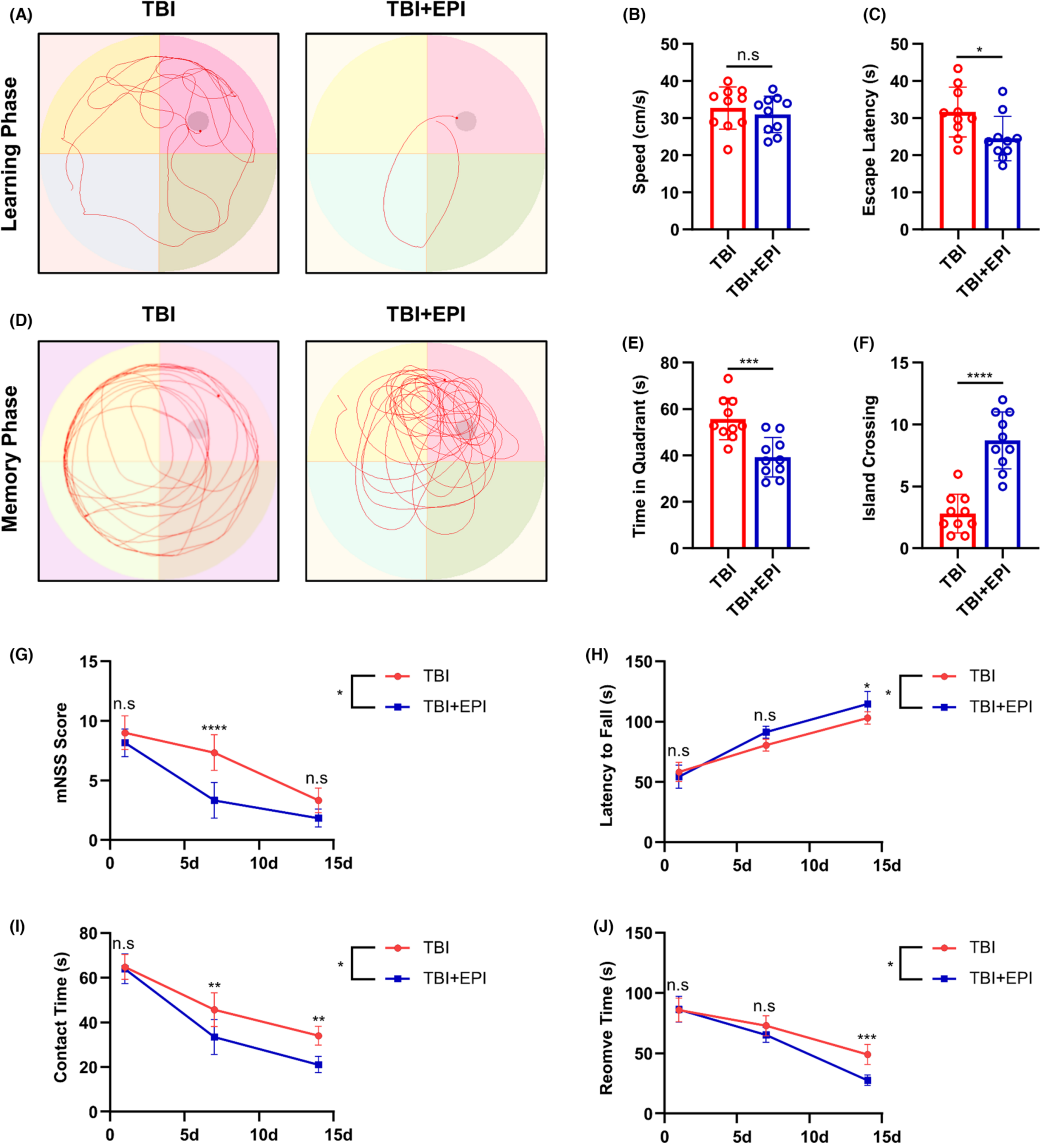
Epicatechin improves neurological function in rats after TBI. (A) Representative results of the Morris water maze during the learning period for TBI rats treated with epicatechin; (B) Quantitative statistics of rat speed; (C) Quantitative statistics of escape latency in rats; (D) Representative results of the Morris water maze during the memory period for TBI rats treated with epicatechin; (E) Quantitative statistics of dwell time in the target quadrant in rats; (F) Quantitative statistics of crossing islets in rats; (G) Quantitative statistics of mNSS scores in rats at 1D, 7D, and 14D after TBI; (H) Quantitative statistics of rod‐turning experiments in rats at 1D, 7D, and 14D after TBI; (I, J) Quantitative statistics of adhesion removal experiments in rats at 1D, 7D, and 14D after TBI. ns, *p* > 0.05; *, *p* ≤ 0.05, **, *p* ≤ 0.01; ***, *p* ≤ 0.001; ****, *p* ≤ 0.0001. The results are presented as mean ± SEM *n* = 6/group.

We investigated the potential protective effects of epicatechin treatment on learning (Figure 3A) and memory (Figure 3D) functions using the Morris water maze. Notably, epicatechin treatment did not affect swimming speed (Figure 3B), but significantly reduced the time to find the platform during the learning phase (Figure 3C), suggesting a beneficial effect on the learning function of rats. Additionally, during the memory phase, rats treated with epicatechin spent more time in the target quadrant (Figure 3E) and crossed the platform more frequently (Figure 3F), indicating that epicatechin treatment facilitated the recovery of learning and memory functions following TBI (Figure 3A–F). The mNSS is an established measure of neurological function in rats, and our results showed that epicatechin‐treated rats exhibited a more rapid recovery of neurological function (Figure 3G). Furthermore, the results of the rotarod test (Figure 3H) and the adhesive‐removal test (Figure 3I,J) supported our hypothesis that epicatechin protects the neurological function of rats after TBI, promoting rapid recovery.

### 3.4 Epicatechin inhibits the activation of microglia in the TBI injury area and reduces uncontrolled neuroinflammation after TBI

**FIGURE 4:**
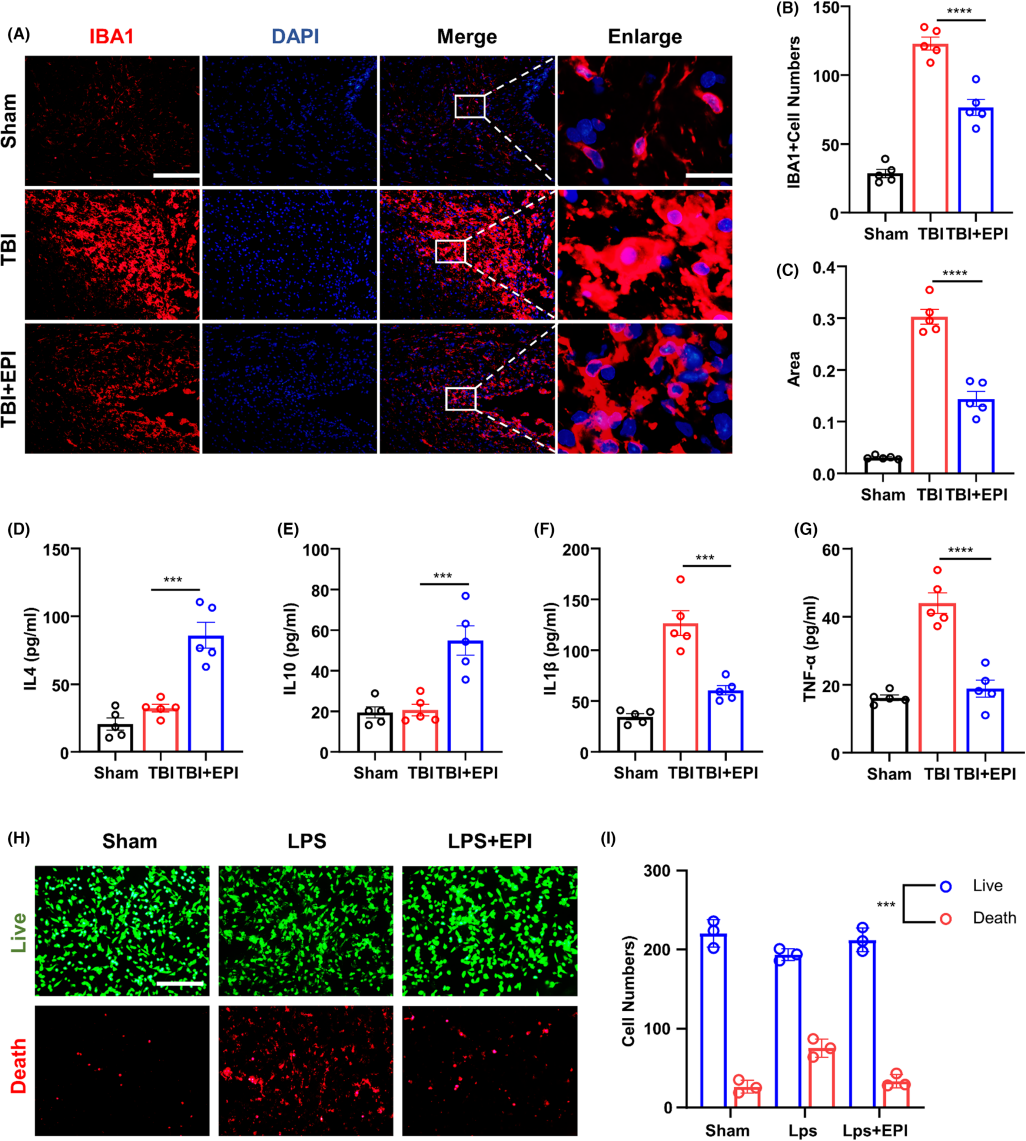
Epicatechin inhibits activation and apoptosis of microglia after TBI. (A) Representative immunofluorescence images of microglia in the injury area after TBI with or without epicatechin treatment; (B) Quantitative analysis of the number of microglia in the injury area; (C) Quantitative analysis of the area of microglia; (D–G) ELISA analysis of the expression levels of IL4, IL10, IL1β, and TNF‐α in a brain tissue homogenate from the injury area; (H, I) Vital cell staining for the anti‐apoptotic ability of LPS‐stimulated primary microglia after epicatechin treatment. Anti‐apoptotic ability of microglia. ns, *p* > 0.05; *, *p* ≤ 0.05, **, *p* ≤ 0.01; ***, *p* ≤ 0.001; ****, *p* ≤ 0.0001. The results are presented as mean ± SEM *n* = 5/group.

The status of microglia is a crucial factor influencing neuroinflammation, and prolonged neuroinflammation can lead to further deterioration of the prognosis of TBI. Using immunofluorescence, we observed a significant reduction in the number of activated microglia after early administration of epicatechin intervention, along with a decrease in cell size and structural complexity, as compared to the group without epicatechin treatment (Figure 4A–C). We then performed an ELISA assay on brain tissue obtained from the injury site and found homogenization and performed ELISA assay on brain tissue obtained from the injury site and found that epicatechin administration led to a significant increase in the levels of anti‐inflammatory cytokines, IL4, and IL10 (Figure 4D,E), and a significant decrease in the levels of pro‐inflammatory cytokines, IL1β, and TNF‐α (Figure 4F,G). Furthermore, in vitro experiments showed that epicatechin effectively reduced the number of apoptotic microglia in LPS‐stimulated microglia and microglia treated with epicatechin after LPS stimulation (Figure 4H,I).

**FIGURE 5:**
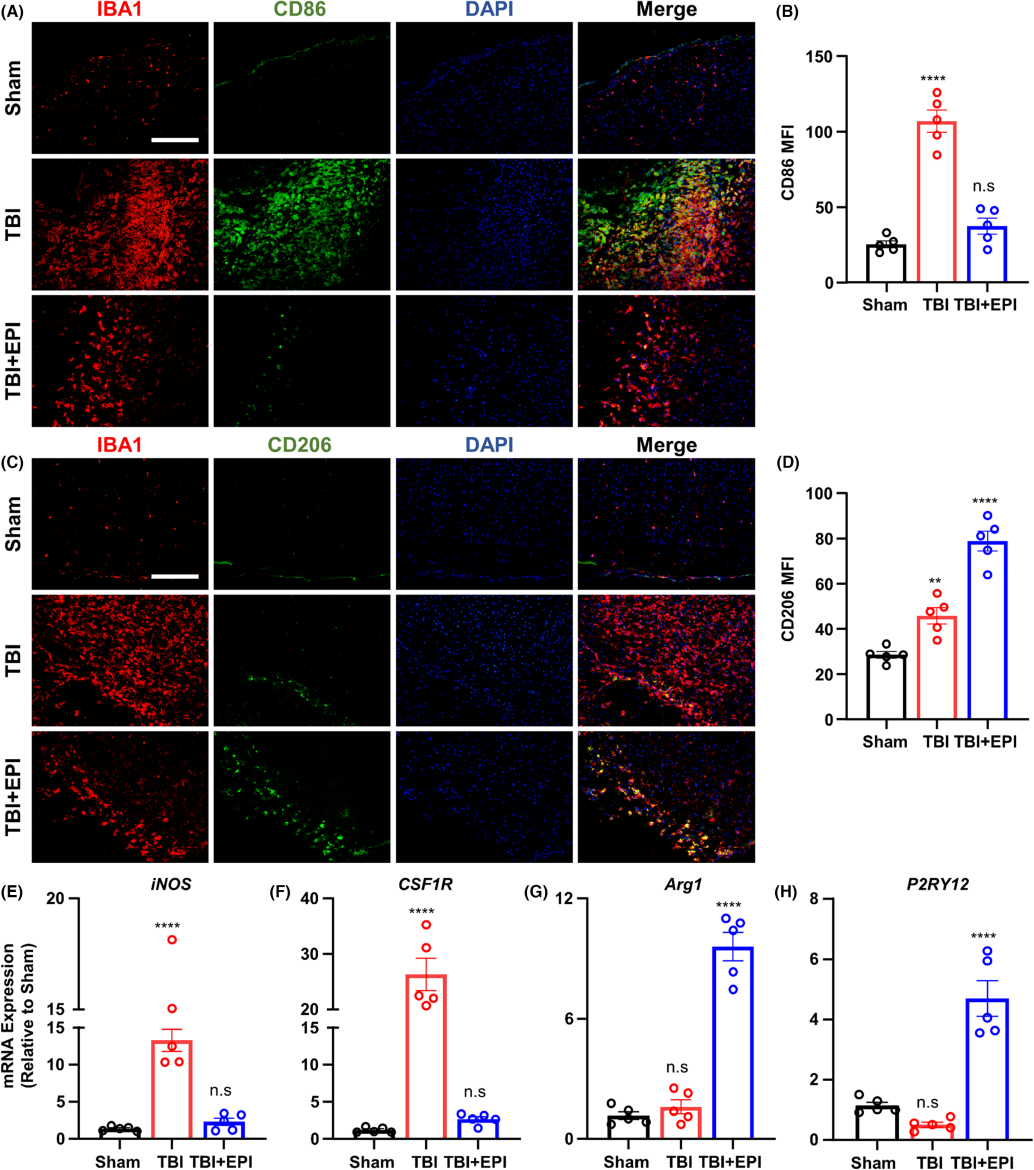
Epigallocatechin regulates the phenotype of microglia infiltrated in the TBI lesion area. (A) Representative fluorescence results of CD86 expression in microglia in the injury zone; (B) Quantitative analysis of the mean fluorescence intensity of CD86; (C) Representative fluorescence images of CD206 expression in microglia in the injury zone; (D) Quantitative analysis of the mean fluorescence intensity of CD86; (E–H) qRT‐PCR detection of iNOS, CSF1R, Arg1, and P2RY12 expression around the injury zone. ns, *p* > 0.05; *, *p* ≤ 0.05, **, *p* ≤ 0.01; ***, *p* ≤ 0.001; ****, *p* ≤ 0.0001. The results are presented as mean ± SEM *n* = 5/group.

CD86 is a marker that indicates microglia's killing function, leading to the amplification of neuroinflammation. Our analysis of the proportion of cells double positive for CD86 and IBA1 in the injury area indicated an extremely pro‐inflammatory state after TBI, which was significantly improved after epicatechin treatment, suppressing overactive microglia (Figure 5A,B). Moreover, we examined the expression of CD206, a marker of the neuroprotective effect of microglia, and found that the administration of epicatechin decreased the number of microglia and increased the expression of CD206, leading to a predominantly neuroprotective function that suppressed inflammation and promoted tissue repair (Figure 5C,D). Microglia in the injury area after epicatechin treatment were dominated predominantly neuroprotective in function, which helped to suppress inflammation and promote tissue repair. Subsequent PCR analyses of brain tissues from the injury area revealed decreased expression of neurotoxic markers such as iNOS and CSF1R (Figure 5E,F) and significantly increased expression of neuroprotective markers such as Arg1 and P2RY12 in response to epicatechin (Figure 5G,H).

To validate our findings, we conducted in vitro experiments with primary microglia, inducing their activation with LPS before administering epicatechin for rescue. First, we examined the optimal concentration of epicatechin for the treatment of microglia inflammation in vitro. After LPS stimulation of microglia, we treated microglial inflammation in six kinds of concentrations: 0 μg/mL, 10 μg/mL, 20 μg/mL, 40 μg/mL, 80 μg/mL, and 160 μg/mL (Figure S1A). We could observe that the inflammatory status of microglia decreased with increasing concentrations of epicatechin treatment (Figure S1C). However, treatment of microglial inflammation at concentrations of 80 μg/mL and 160 μg/mL did not significantly improve compared to 40 μg/mL, but resulted in a decrease in microglia viability (Figure S1B,C). Therefore, we chose 40 μg/mL as the treatment concentration in our in vitro experiments. We then examined the expression of cytokines in microglia medium after 40 μg/mL epicatechin treatment. The concentrations of IL1β and TNF‐α decreased after epicatechin treatment (Figure S1D,E), and IL4 and IL10 increased after epicatechin treatment (Figure S1F,G).

**FIGURE 6:**
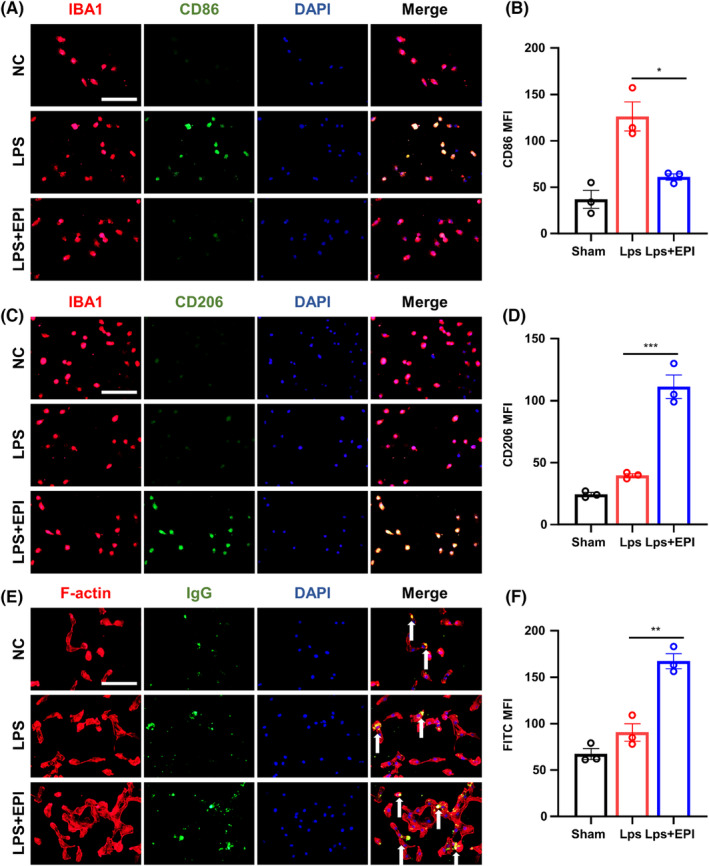
Epicatechin reduces the inflammation level of microglia after LPS stimulation. (A) Representative fluorescence images of CD86 expression in microglia after LPS stimulation with or without epicatechin treatment; (B) Quantitative analysis of the mean fluorescence intensity of CD86; (C) Representative fluorescence images of CD206 expression in microglia; (D) Quantitative analysis of the mean fluorescence intensity of CD206; (E) In vitro microglia phagocytosis assay; (F) Quantitative analysis of the mean fluorescence intensity of IgG‐FITC phagocytosed by the microglia. ns, *p* > 0.05; *, *p* ≤ 0.05, **, *p* ≤ 0.01; ***, *p* ≤ 0.001; ****, *p* ≤ 0.0001. The results are presented as mean ± SEM *n* = 3/group.

We observed a decrease in the expression of the toxicity marker CD86 and an increase in the expression of the neuroprotective marker CD206, consistent with our in vivo results (Figure 6A–D). Additionally, we used green fluorescent IgG to detect antigen phagocytosis in microglia. Epicatechin administration enhanced cell phagocytosis and promoted the quiescence of inflammation (Figure 6E,F). Taken together, our in vivo and in vitro results demonstrate that epicatechin significantly inhibits excessive neuroinflammation and suppresses apoptosis in the CNS after TBI.

### 3.5 Epicatechin potentially exerts therapeutic functions through AKT‐P53/CREB pathway

**FIGURE 7:**
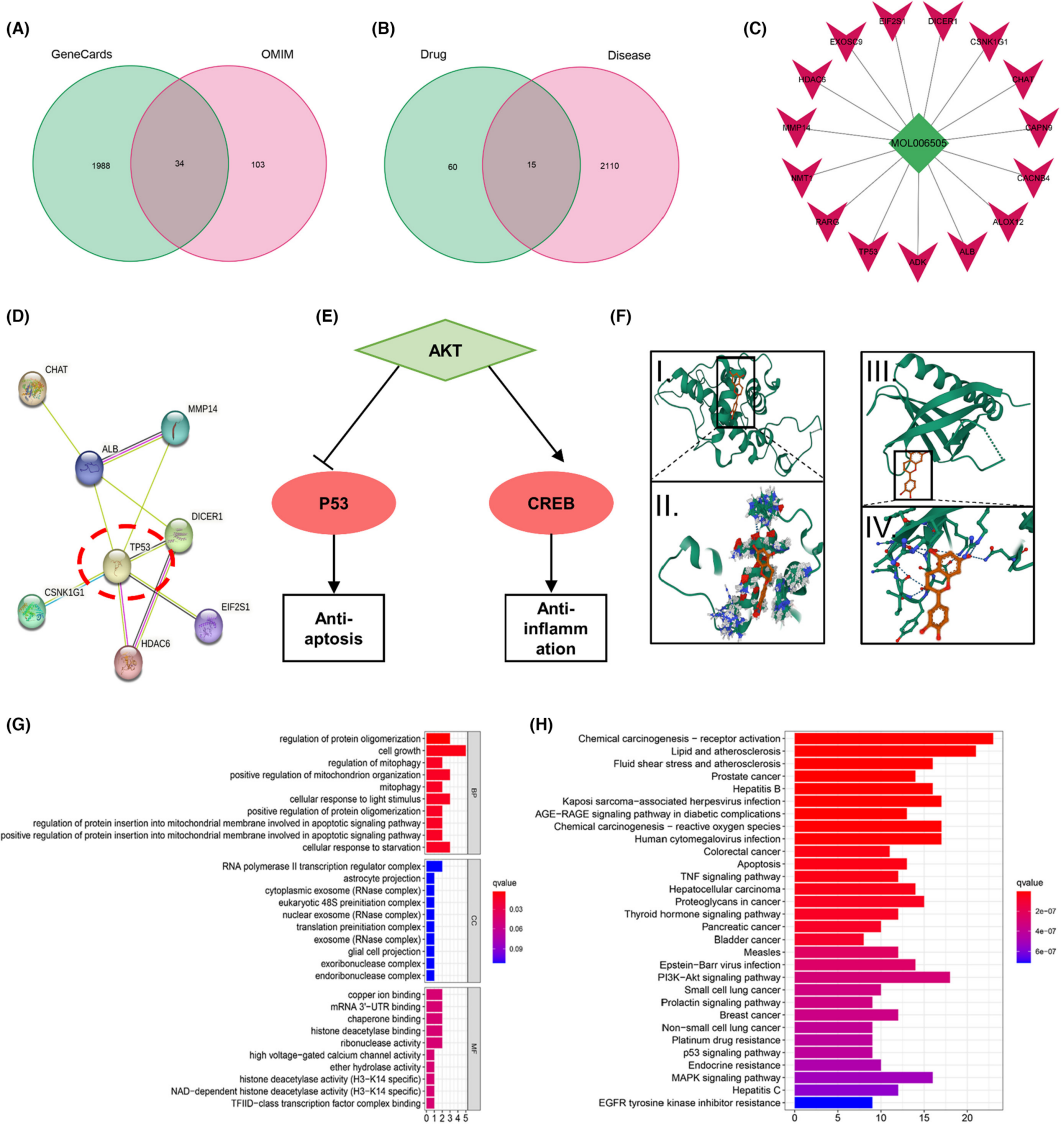
Network pharmacology reveals the molecular mechanism of epicatechin in TBI. (A) The Genepirate and OMIM databases were searched using “Traumatic brain injury” as the keyword, resulting in 2125 targets after concatenation; (B) Intersection of the 75 epicatechin targets and 2125 TBI disease targets using Perl software resulted in 15 overlapping targets specific to epicatechin; (C) Epicatechin (green diamond) and 15 intersecting target genes (red triangles) are shown; (D) Protein interaction network of key targets; (E) AKT‐related pathway pattern map; (F) Molecular docking pattern map of epicatechin and pathway; (G) GO analysis of related biological functions; (H) KEGG analysis of related pathways involved.

To investigate the potential molecular biological mechanism of epicatechin in the treatment of TBI, we conducted a comprehensive analysis using the GENECARDS and OMIM databases. A search for epicatechin with the keyword “Traumatic brain injury” yielded a total of 2125 TBI‐related targets (Figure 7A). Subsequently, we obtained the molecular structure of epicatechin and mapped it to gene symbols, resulting in the identification of 75 targets associated with epicatechin. Utilizing Perl software, we performed an intersection analysis between these 75 potential therapeutic targets and the 2125 TBI disease targets, leading to the identification of 15 common targets (Figure 7B,C, Table S2). Analysis of the PPI network and the corresponding bar chart for the 15 intersecting targets revealed that TP53, ALB, DICER1, HDAC6, and MMP14 occupy central positions within the network, characterized by the highest number of neighboring nodes. These findings suggest that these genes, including TP53, ALB, DICER1, HDAC6, and MMP14, may serve as the core genes targeted by epicatechin for TBI control.

Further investigation of the KEGG database indicated that the AKT pathway might be an upstream regulator of TP53 expression. Interestingly, we also observed a close association of CREB, a molecule within the same pathway (Figure 7E). Our suspicion was supported by the molecular docking analysis, which demonstrated the structural compatibility between epicatechin and TP53/AKT. Notably, the calculated binding energies for TP53 and AKT with epicatechin were −80.971 and −6.225 kcal/mol, respectively, indicating a highly stable binding interaction (Figure 7F). Gene ontology (GO) analysis of the results suggested the involvement of epicatechin in biological processes such as the regulation of protein oligomerization and cell growth (Figure 7G, Table S3). Additionally, the KEGG analysis indicated that epicatechin may exert its effects through the AKT‐P53/CREB pathway (Figure 7H, Table S4).

### 3.6 Inhibition of AKT pathway reduces the regulatory effect of epicatechin on microglia

**FIGURE 8:**
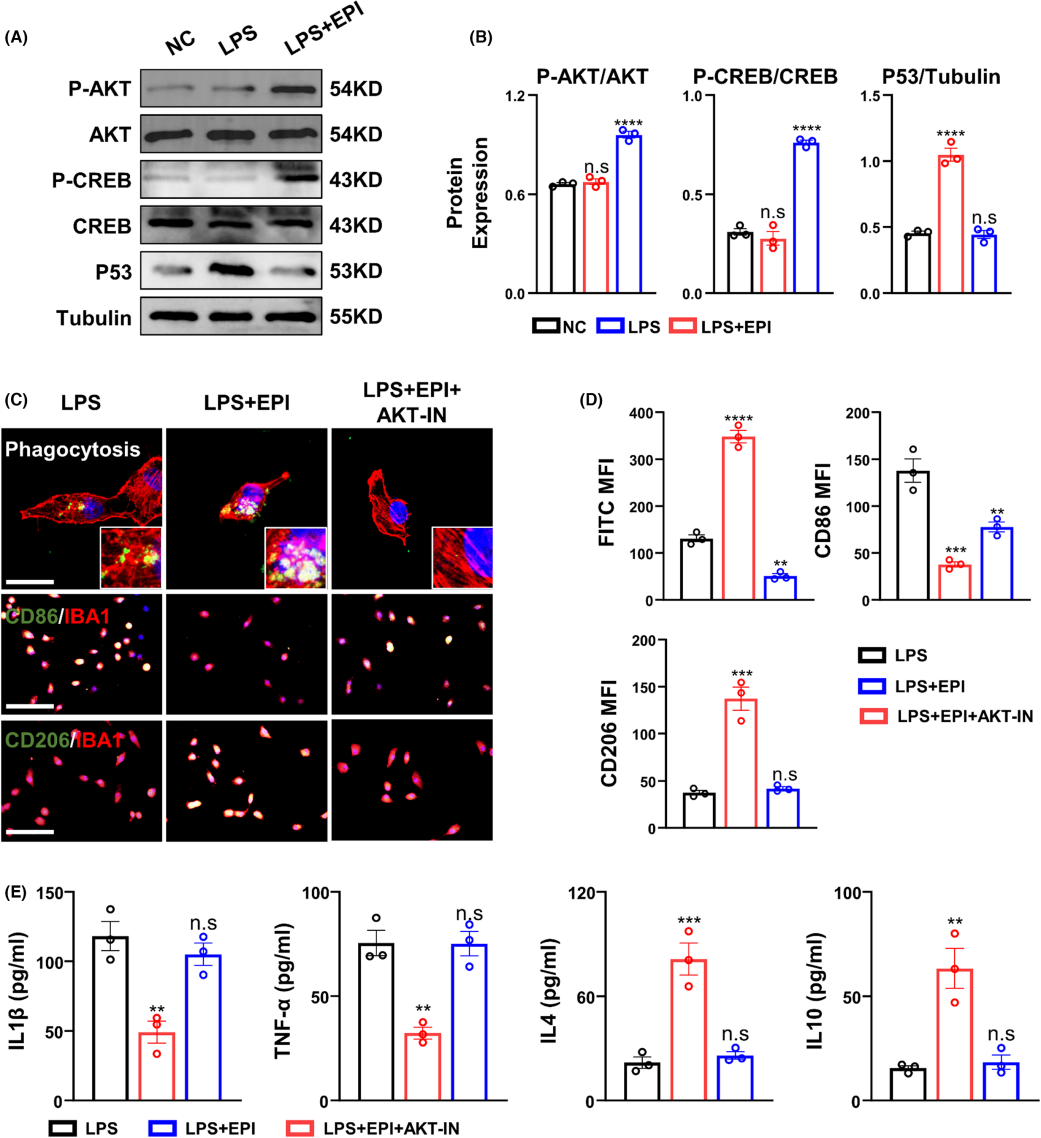
Epicatechin exerts neuroprotective function through AKT‐related pathway. (A) Representative Western blot results for the expression of AKT pathway‐related molecules in brain tissue homogenates; (B) Quantitative analysis of the Western blot results; (C) In vitro stimulation of microglia with AKT inhibitors followed by cell phagocytosis and expression of CD86 and CD206; (D) Quantitative analysis of the mean fluorescence intensity of IgG‐FITC, CD86, and CD206; (E) ELISA results for the expression levels of IL4, IL10, IL1β, and TNF‐α in the medium of cultured microglia. ns, *p* > 0.05; *, *p* ≤ 0.05, **, *p* ≤ 0.01; ***, *p* ≤ 0.001; ****, *p* ≤ 0.0001. The results are presented as mean ± SEM *n* = 3/group.

We first examined AKT pathway‐related molecules obtained from network pharmacological analysis using Western blot, and observed a significant increase in the expression levels of P‐AKT and P‐CREB after epicatechin administration, while P53 levels were decreased (Figure 8A,B). Upon treatment with an AKT pathway inhibitor, we evaluated the ability of cells upon epicatechin administration with AKT inhibitor AKT‐IN, leading to a re‐increase in CD86 expression, and a suppression in the level of CD206 expression (Figure 8C,D).

Furthermore, ELISA assay of the supernatant collected from microglia switched to basal medium for 24 h showed an increase in the expression of pro‐inflammatory cytokines IL1β and TNF‐α after AKT pathway inhibition, while the expression levels of IL4 and IL10 decreased (Figure 8E).

### 3.7 Inhibition of AKT pathway reduces the neuroprotective effect of epicatechin on TBI rats

**FIGURE 9:**
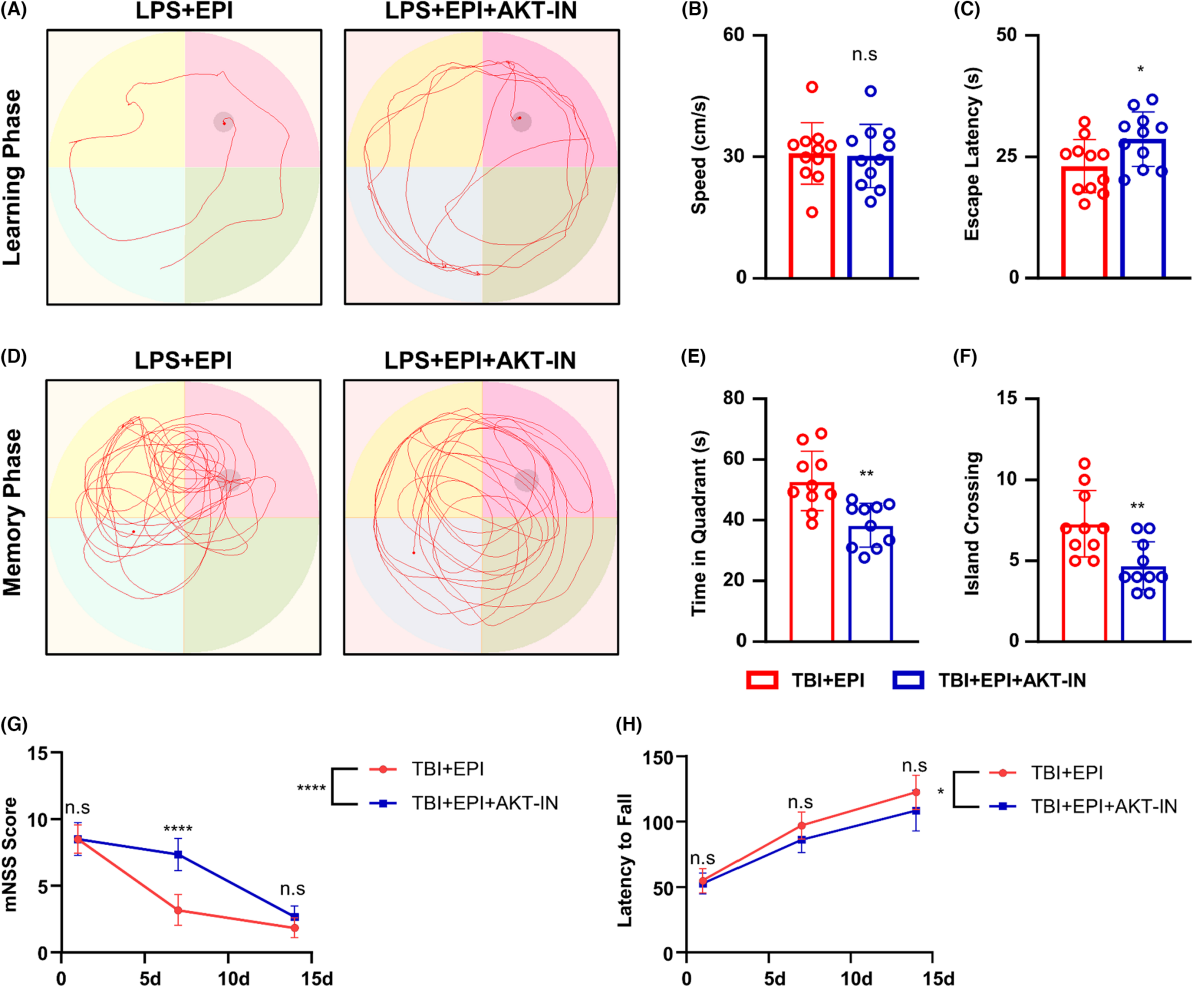
Reduced therapeutic effect of epicatechin after inhibition of AKT pathway. (A) Representative results of the Morris water maze test during the learning period in TBI rats; (B) Quantitative statistics of swimming speed in rats; (C) Quantitative statistics of escape latency in rats; (D) Representative results of Morris water maze test during the memory period in TBI rats; (E) Quantitative statistics of dwell time in the target quadrant in rats; (F) Quantitative statistics of the number of platform crossings in rats; (G) Quantitative statistics of the mNSS scores in rats at 1D, 7D, and 14D after TBI; (H) The quantitative statistics of the rod‐turning experiment in rats at 1D, 7D, and 14D after TBI. ns, *p* > 0.05; *, *p* ≤ 0.05, **, *p* ≤ 0.01; ***, *p* ≤ 0.001; ****, *p* ≤ 0.0001. The results are presented as mean ± SEM (A–F) *n* = 10/group, (G–H) *n* = 6/group.

After blocking the AKT pathway signal in TBI rats with an AKT inhibitor, we evaluated the learning and memory ability and neurological function of epicatechin‐treated TBI rats. The results showed that the neuroprotective effect of epicatechin on neurological function was significantly blocked with an AKT inhibitor, and the Morris water maze showed a prolongation in the learning phase and the time to find the island in TBI rats (Figure 9A–C). During the memory phase, the TBI rats showed a shortened stay in the target quadrant and a reduced number of island crossing times (Figure 9D–F). The results of the final mNSS score (Figure 9G) and the rotarod test (Figure 9H) corroborate our idea that the use of AKT inhibitors blocked the neuroprotective function of epicatechin.

## 4 DISCUSSION

Despite extensive research into the molecular biology of traumatic brain injury (TBI), treatment options for this condition have not significantly progressed. There is an urgent need for effective medications to reduce neurological damage and improve outcomes for patients.^4^ To address this need, we investigated the potential of epicatechin as a treatment for TBI using a rat model. Our study revealed several key findings. First, we observed that epicatechin significantly improved neurological function and reduced inflammatory response following TBI. Second, we identified that epicatechin may alleviate secondary inflammatory injury via the AKT‐P53/CREB signaling pathway. Finally, we found that inhibiting the AKT signaling pathway blocked the neuroprotective effects of epicatechin. Our results suggest that epicatechin has a potential therapeutic in regulating the inflammatory response to TBI via the AKT‐P53/CREB signaling pathway.

Chronic pathogenic processes such as glutamate excitotoxicity, mitochondrial dysfunction, DNA damage, oxidative stress, and inflammation are among the causes of secondary brain injury following TBI.^17^ Research has shown that persistent neuroinflammation is responsible for the decline in function following a TBI.^18^, ^19^ An appropriate inflammatory response can help repair the damage and promote neuronal remodeling, while an excessive inflammatory response can result in persistent neuronal damage, oxidative stress, and brain edema. Various cells within the CNS are activated by neuronal injury, including microglia, astrocytes, and cerebrovascular endothelial cells.^17^ When inflammation occurs, immune cells are recruited to the area of injury by releasing chemokines from damaged neuronal tissue. In turn, microglia are rapidly activated in the CNS, causing the release of inflammatory mediators such as IL‐1β, TNF‐α, IL‐6, and CCL2 into brain tissue, thus triggering an inflammatory response. Increased pro‐inflammatory factor levels cause neuronal deformation and structural and functional brain abnormalities by promoting matrix metalloproteinase production, inducing neuronal death and rupturing the blood–brain barrier.^20^, ^21^

With increasing research on neuroinflammation after brain injury, it has been found that microglia can exhibit different functions in different environments. The secretion of a variety of cytokines, chemokines, and complement cascade components by microglia can impact axonal extension and functional rebuilding. This can be harmful to the survival of neurons and oligodendrocytes after injury and affect axonal extension and uncontrollable immune responses that promote the development of neurological and neuropsychiatric diseases linked to TBI.^20^ However, microglia can also enhance phagocytic clearance, maintain internal environmental homeostasis, upregulate neurotrophic factors to counteract neuronal injury and promote regeneration, and act as neuroprotective agents. Therefore, the regulation of microglia infiltrating the injury zone is a hot area of research on TBI. In the present study, we found that epicatechin, when administered at a dose of 150 μg/100 g, significantly reduced microglia‐mediated neuroinflammation in TBI rats. We observed an increase in CD206 and Arg1 expression in microglia and a decrease in CD86 and iNOS transcript levels. Consistent results were observed in LPS‐activated microglia cultured in vitro. These results indicate that epicatechin can prevent the overactivation of microglia and regulate the phenotype of microglia in the TBI model. Thus, it can help suppress neuroinflammation and repair neurological function in TBI rats.^22^, ^23^, ^24^

The P13K–AKT signaling pathway has gained significant attention in recent years due to its involvement in regulating various physiological and pathological processes, including cell development, differentiation, stress, and inflammation. Evidence suggests that TBI also activates P13K–AKT pathway,^25^ which shares connections with many inflammatory regulatory mechanisms. Our study utilized network pharmacology with Western blot analysis to reveal that TBI significantly increased P53 protein expression, which caused rapid cell death in the injury site. In contrast, epicatechin exerted neuroprotective effects by promoting phosphorylation of AKT and its downstream CREB, inhibiting the expression of P53, and enhancing anti‐inflammatory effects. Our results showed that epicatechin treatment enhanced the phosphorylation of AKT, leading to inhibition of the P53 pathway and enhanced CREB phosphorylation, thereby inhibiting apoptosis and reducing the synthesis of inflammatory cytokines in the injury area.^26^, ^27^

While the protective effect of epicatechin on TBI involves complex molecular mechanisms, recent studies suggest that epicatechin attenuates inflammatory responses in microglia by inhibiting the p38/MAPK/NF‐κB signaling pathway. Therefore, our study aimed to investigate whether epicatechin attenuates secondary brain injury after TBI through the AKT‐P53/CREB signaling pathway. However, further analysis is needed to fully understand the specific mechanisms by which epicatechin alleviates secondary neurological impairment in TBI patients and promotes the recovery of learning and memory function. This remains the focus of our future work.

## 5 CONCLUSION

In conclusion, our study demonstrates that epicatechin, extracted from a traditional Chinese herb, exhibits anti‐inflammatory and neuroprotective effects following brain injury, possibly through the regulation of the AKT‐P53/CREB signaling pathway. Epicatechin has promising clinical translational potential as a therapeutic agent for TBI, as it promotes AKT and CREB phosphorylation while inhibiting p53, thereby suppressing neuroinflammation and promoting neurological recovery after TBI (Figure S2).

## AUTHOR CONTRIBUTIONS

Conceptualization: QQL, RJ, and PPG. Methodology: ZHW and ZCL. Validation: ZCL, ZHW, YXC, and CXW. Formal analysis: ZCL and ZHW. Writing—original draft preparation: ZCL and ZHW. Writing—review and editing: QQL, RJ, and PPG. Visualization, ZHW, ZCL, YXC, and CXW. Supervision: QQL, RJ, and PPG. All authors have read and agreed to the published version of the manuscript.

## FUNDING INFORMATION

This research was funded by the National Natural Science Foundation of China (82271415), Postgraduate Research and Practice Innovation Program of Jiangsu Province (KYCX22‐3364), and Nantong Natural Science Foundation (JC12022063).

## CONFLICT OF INTEREST STATEMENT

The authors declare no conflict of interest.

## Supporting information


Table S1

Table S2

Table S3

Table S4

Figures S1‐S2

## Data Availability

Data will be available upon reasonable request.
