# Supplementary material for: Targeting the AKT‐P53/CREB pathway with epicatechin for improved prognosis of traumatic brain injury
Source: CNS Neurosci Ther. 2023 Jul 18;30(2):e14364. doi: 10.1111/cns.14364 (PMC10848092; doi:10.1111/cns.14364)
Supplement: Supplementary file 5 — Figures S1‐S2 [file CNS-30-e14364-s003.docx]

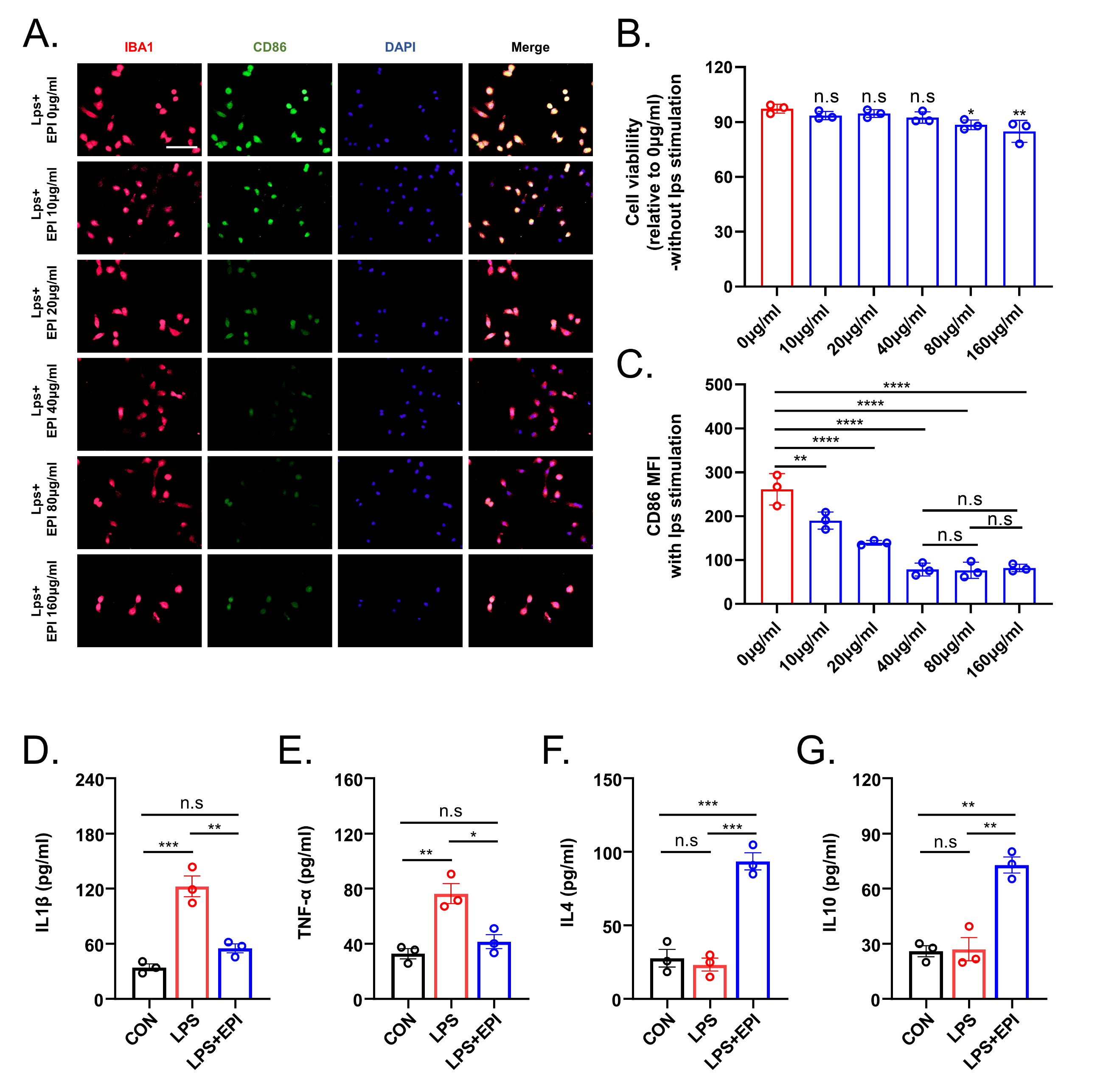


**Supplementary Figure 1. Exploration of the most suitable therapeutic concentration of epicatechin in vitro.** (A) Representative results of CD86 immunofluorescence after treatment of microglia with different concentrations of epicatechin after lps stimulation; (B) Changes in cell viability after treatment of microglia with different concentrations of epicatechin; (C) Quantitative statistics for the mean fluorescence intensity of CD86 in A; (D-G) The concentrations of IL1β, TNF-α, IL4 and IL10 in the culture medium were measured after treatment of lps-stimulated microglia with epicatechin at a concentration of 40 μg/ml. n.s, P＞0.05; *, P≤0.05, **, P≤0.01; ***, P≤0.001; ****, P≤0.0001. The results are presented as mean ± S.D. n = 3/group


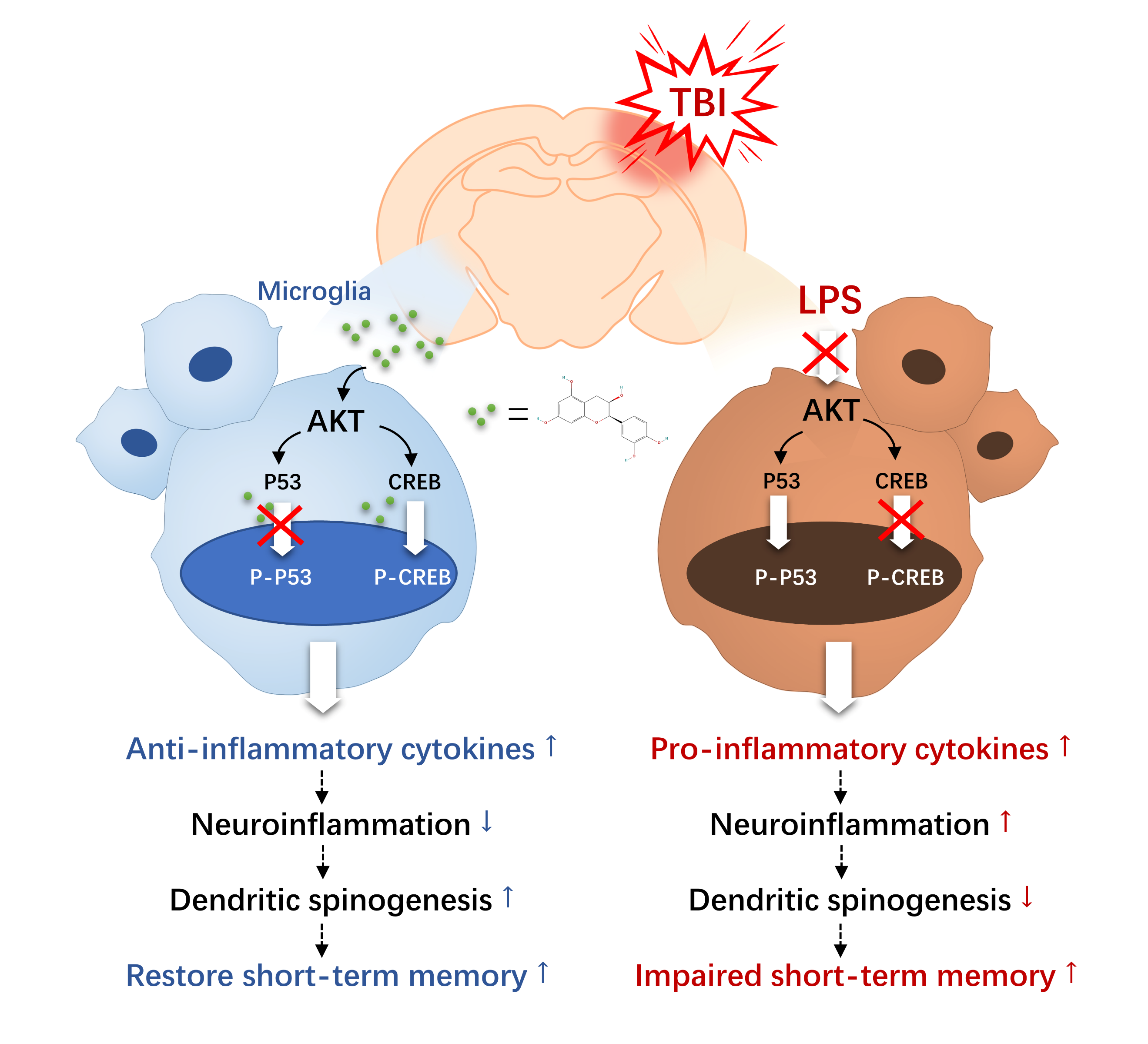


**Supplementary Figure 2. Graphical abstract.** Epicatechin has good clinical translational prospects as a potential therapeutic drug for TBI because it can promote phosphorylation of AKT and CREB while inhibiting P53, thereby suppressing neuroinflammation and promoting neurological recovery after TBI.
